# Inhibition of Tolaasin Cytotoxicity Causing Brown Blotch Disease in Cultivated Mushrooms Using Tolaasin Inhibitory Factors

**DOI:** 10.3390/toxins15010066

**Published:** 2023-01-12

**Authors:** Yeong-Bae Yun, Kwang-Hyun Cho, Young-Kee Kim

**Affiliations:** 1 Department of Environmental and Biological Chemistry, Chungbuk National University, Cheongju 28644, Republic of Korea; 2 Forest Medicinal Resources Research Center, National Institute of Forest Science, Yeongju 36040, Republic of Korea

**Keywords:** artificial lipid bilayer, oyster mushroom (*Pleurotus ostreatus*), pore-forming peptide toxin, *Pseudomonas tolaasii*, tolaasin

## Abstract

Tolaasin, a pore-forming bacterial peptide toxin secreted by *Pseudomonas tolaasii*, causes brown blotch disease in cultivated mushrooms by forming membrane pores and collapsing the membrane structures. Tolaasin is a lipodepsipeptide, MW 1985, and pore formation by tolaasin molecules is accomplished by hydrophobic interactions and multimerizations. Compounds that inhibit tolaasin toxicity have been isolated from various food additives. Food detergents, sucrose esters of fatty acids, and polyglycerol esters of fatty acids can effectively inhibit tolaasin cytotoxicity. These chemicals, named tolaasin-inhibitory factors (TIF), were effective at concentrations ranging from 10^−4^ to 10^−5^ M. The most effective compound, TIF 16, inhibited tolaasin-induced hemolysis independent of temperature and pH, while tolaasin toxicity increased at higher temperatures. When TIF 16 was added to tolaasin-pretreated erythrocytes, the cytotoxic activity of tolaasin immediately stopped, and no further hemolysis was observed. In the artificial lipid bilayer, the single-channel activity of the tolaasin channel was completely and irreversibly blocked by TIF 16. When TIF 16 was sprayed onto pathogen-treated oyster mushrooms growing on the shelves of cultivation houses, the development of disease was completely suppressed, and normal growth of oyster mushrooms was observed. Furthermore, the treatment with TIF 16 did not show any adverse effect on the growth of oyster mushrooms. These results indicate that TIF 16 is a good candidate for the biochemical control of brown blotch disease.

## 1. Introduction

*Pseudomonas tolaasii* is a pathogen that causes brown blotch disease in cultivated mushrooms [1]. The bacterial causative agent, tolaasin, is a pore-forming lipodepsipeptide toxin [2]. Sources of *P. tolaasii* contamination in cultivation houses include irrigation systems using underground water and air-borne contamination. In particular, the disease frequently develops under poor growth conditions, such as during cool periods (early spring), and when large amounts of rainfall cause humidity [3].

The tolaasin peptide comprises 18 amino acids with β-hydroxyoctanoic acid at the N-terminus. It exhibits surfactant activity as an amphiphilic peptide, with intermolecular hydrophilic and hydrophobic groups. The surface tension of the mushroom is lowered by tolaasin molecules, allowing pathogenic bacteria to infect the mushroom tissue [3]. The mode of action of tolaasin in pathogen infection is similar to that of other peptide toxins, such as ZorO toxin, melittin, and corpeptin [4]. Briefly, tolaasin molecules bind to the cell membrane surface and become inserted in the membrane, forming pores via molecular multimerization. Tolaasin pores collapse the membrane structure of mushroom cells by disturbing the cellular osmotic pressure [5,6]. Through this mechanism, tolaasin molecules are also able to lyse the membrane of erythrocytes; therefore, tolaasin toxicity can be demonstrated by measuring tolaasin-induced hemolysis.

Once brown blotch disease occurs amongst cultivated mushrooms, their continued cultivation becomes very difficult since the disease can spread rapidly throughout the entire cultivation area. Sterilization of agricultural underground water and disinfection of cultivation facilities have been attempted to prevent brown blotch disease [7,8]; however, the disease is not easily suppressed. Antagonistic microbes have shown partially satisfactory results when used for the biological treatment of brown blotch disease [9]. Bacteriophages specific to the pathogens of *P. tolaasii* have an excellent effect, completely removing the disease. However, phage-resistant mutant pathogens frequently occur; further studies on these mutants would be helpful for improving phage therapy methods. Antibiotics successfully suppress brown blotch disease; however, they are not considered applicable to mushroom cultivation since mushrooms are fresh foods [10].

Considering the significant economic losses caused by the disease, research on brown blotch disease has been insufficient, and to date, no suitable methods have been developed to control the disease. We believe that an effective approach for the treatment or prevention of brown blotch disease entails disrupting or inhibiting the multimerization of tolaasin molecules to avert the formation of the pore structure and destroy tolaasin cytotoxicity [5]. Tolaasin multimerization may occur through hydrophobic interactions among the molecules, meaning detergents may be used to interrupt the forces stabilizing the multimer. In our previous studies, it was found that various food additives, including detergents, were able to suppress the disease; therefore, they were named tolaasin-inhibitory factors (TIFs).

The inhibitory effects of TIFs on tolaasin-induced hemolysis have also been investigated [11]. Polyglycerol and sucrose esters of fatty acids effectively inhibited tolaasin cytotoxicity. Among them, TIF 16 (polyglycerol fatty acid ester, MW 1003) showed the most potent inhibitory effect on tolaasin-induced cytotoxicity [11]. The TIFs were stable and remained active for several months under anaerobic conditions.

In this study, we investigated the characteristics of TIF 16 to better understand the role of tolaasin peptides in disease and to develop a possible candidate for disease treatment. We also examined the effects of TIF 16 on tolaasin channel activity in the lipid bilayer membrane. The inhibitory effect of TIF 16 on tolaasin, and hence on brown blotch disease, was evaluated through the on-farm cultivation of oyster mushrooms.

## 2. Results

### 2.1. Temperature and pH Dependence of TIF 16 Effects

Various food additives were screened for their potential as tolaasin toxicity inhibitory factors (TIFs) [11]. The most effective materials were various food emulsifying agents, which seemingly inhibit the pore formation of tolaasin molecules through hydrophobic interactions. These TIFs are unsaturated carbon compounds that are sensitive to air exposure and light irradiation, but stable under anaerobic conditions. Although the inhibitory effect of TIFs was found to be stable under various temperature conditions, the cytotoxicity of tolaasin was temperature- and pH-dependent [12]. Therefore, we investigated the inhibitory effects of TIF 16 under various temperature and pH conditions.

**Figure 1:**
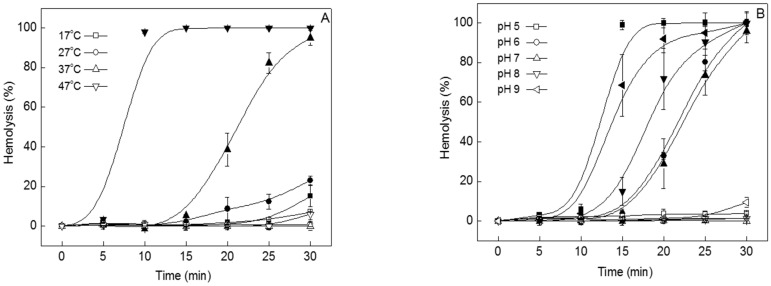
Effect of TIF 16 at various temperatures and under different pH conditions. TIF 16 was added to the tolaasin solution with a final concentration of 10^−5^ M. Filled symbol: tolaasin. Open symbol: tolaasin+TIF 16. (**A**) Temperature-dependent hemolytic activities of tolaasin at temperatures from 17 to 47 °C. (**B**) pH-dependence of hemolysis measured at pH levels from 5 to 9.

One HU of tolaasin was added to the erythrocyte solution, which was heated at various temperatures: 17, 27, 37, and 47 °C (Figure 1A). At 17 and 27 °C, tolaasin-induced hemolysis was less than 20% complete. At the higher temperatures, hemolysis was 100% complete, and the time to its completion decreased with increasing temperature; at 37 °C, hemolysis was 100% complete within 30 min, and at 47 °C, within 10 min (Figure 1A, filled symbols). The rate at which hemolysis occurs can be expressed as T_50_, i.e., the time to reach 50% hemolysis. While the T_50_ values at 17 and 27 °C could not be obtained, T_50_ at 37 and 47 °C was measured as 21.1 and 7.4 min, respectively. When TIF 16 was added to the reaction solution, tolaasin-induced hemolysis was substantially inhibited, with less than 10% hemolysis completion, regardless of the temperature conditions (Figure 1A, open symbol). Therefore, TIF 16 effectively suppressed tolaasin-induced hemolysis under a range of temperature conditions.

The inhibitory effect of TIF 16 was measured under various pH conditions. The pH of buffer solution was adjusted in the range of 5–9 and tolaasin-induced hemolysis was measured at each pH (Figure 1B, filled symbol). At pH 5, erythrocytes were highly unstable and hemolysis occurred rapidly, even in the absence of tolaasin, meaning that at this pH, hemolysis is independent of tolaasin action. No pH effects could be observed at pH values 6–9. At pH 6 and 7, 100% complete tolaasin-induced hemolysis was observed after 30 min. In more alkaline conditions, pH 8 and 9, the tolaasin-induced hemolysis was completed even faster. Yet, in the presence of TIF 16, tolaasin-induced hemolysis was nearly completely suppressed, regardless of the above pH conditions, with hemolysis completion measured at less than 5% (Figure 1B, open symbol).

### 2.2. Effect of TIF 16 on Pretreated Tolaasin Hemolysis

**Figure 2:**
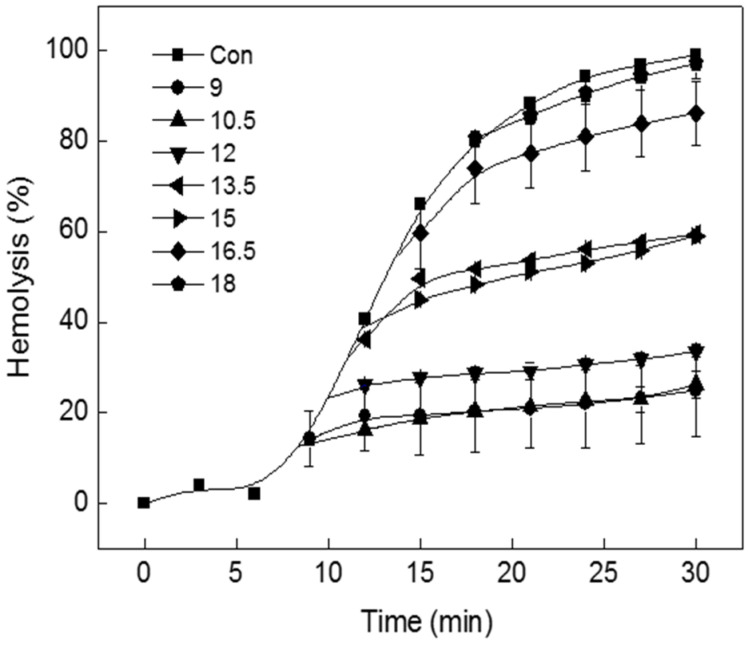
Effect of TIF 16 at various phases of hemolysis. Symbols: the times of TIF 16 addition during hemolysis. TIF 16 was treated at 1.5 min intervals from 9 min after incubation. The final concentration of TIF 16 was 10^−5^ M.

When 1 HU of tolaasin peptide was added to the erythrocyte solution, hemolysis started approximately 10 min after addition. Pre-incubation is required for membrane insertion of tolaasin peptide and for molecular multimerization to form pores. To investigate the inhibition of pre-incubated tolaasin by TIF 16 treatment, the inhibitory effect of TIF 16 was measured when added at different time points (9, 12, 15, 18, 21, 24, 27, and 30 min). To do so, TIF 16 (10^−4^ M) was added to the erythrocyte solution at 1.5 min intervals beginning 9 min after adding tolaasin. In comparison with the control experiment, at the first time point of TIF 16 addition, it immediately suppressed hemolysis such that no further hemolysis was observed (Figure 2); when hemolysis had progressed to 20% or less, further hemolysis was completely suppressed by TIF 16; when hemolysis had progressed to between 20% and 60%, a delay in hemolysis was observed; and when hemolysis had progressed to greater than 60%, the inhibitory effect of TIF 16 was not observed.

### 2.3. Irreversible Binding of TIF 16 to Erythrocyte Membrane

**Figure 3:**
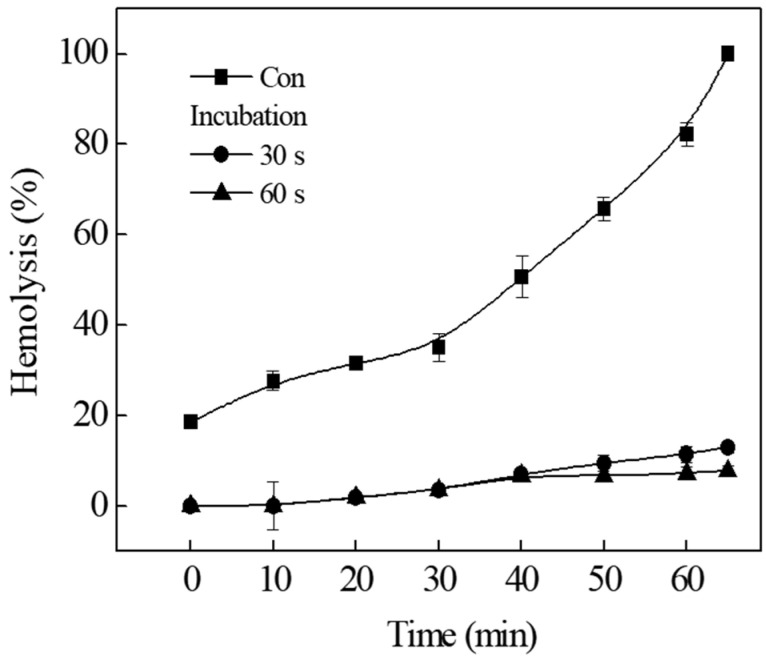
Irreversible binding of TIF 16. Tolaasin was added and washed after 9 min (Con). TIF 16 was added after 9 min of tolaasin addition, and unbound molecules of tolaasin and TIF 16 were washed out after 30 (●) and 60 (▲) s incubations. The washed RBCs were re-suspended with fresh buffer solution.

To investigate whether the binding of TIF 16 to tolaasin-treated erythrocytes was reversible, the hemolytic activity was measured after tolaasin-treated erythrocytes were washed with fresh buffer solution (Figure 3, Con). Erythrocytes were pre-incubated with tolaasin for 5 min and washed with fresh buffer, followed by the addition of TIF 16 (10^−5^ M) to the reaction solution. After incubation for 30 and 60 s, the erythrocytes were centrifuged and the supernatant containing non-bound TIF 16 was removed. The precipitated erythrocytes were re-suspended in fresh buffer solution, and the hemolytic activity obtained with membrane-bound tolaasins was measured. When TIF 16 was absent from the reaction solution, re-suspended erythrocytes were destroyed in a time-dependent manner (Figure 3, Con). The slow increase in hemolytic activity was due to the action of tolaasin molecules already bound to the cytoplasmic membrane. As the unbound tolaasin molecules were removed by centrifugation, there was no additional membrane insertion of tolaasin. However, when the reaction solutions to which TIF 16 was added were incubated for 30 and 60 s, the slow increase in hemolysis was almost completely suppressed. Since the unbound TIF 16 molecules were washed out, membrane-bound TIF 16 molecules inhibited tolaasin cytotoxicity for longer than 60 min. These results indicate that membrane binding of TIF 16 is irreversible.

### 2.4. Inhibition of Tolaasin Channel by TIF 16

**Figure 4:**
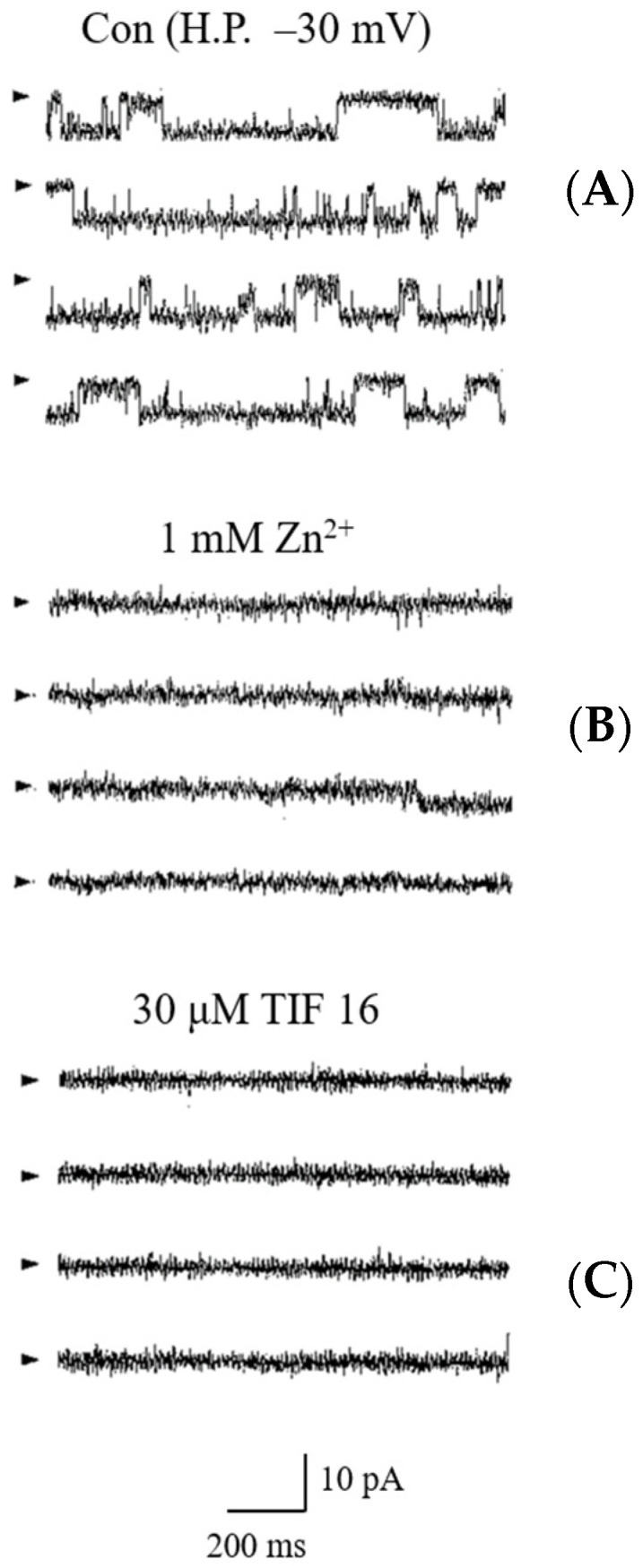
Inhibitory effects of Zn^2+^ and TIF 16 on the tolaasin channel. (**A**) Continuous recordings of the tolaasin channel. Additions of Zn^2+^ (**B**) or TIF 16 (**C**) in symmetric solution containing 100 mM KCl. Arrows (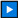) and H.P. mean closed state and holding potential, respectively.

We have shown that tolaasin molecules form ion channels in artificial lipid membranes that are inhibited by Zn^2+^ [5]. Since TIF 16 has an antagonistic effect on tolaasin peptides, it can block the channel activity formed by tolaasin molecules. Tolaasin channels were formed in the lipid bilayer (Figure 4). The channel current increased linearly as the membrane voltage increased, similar to a previous result [5]. The channel exhibited a linear current–voltage relationship. The unit conductance of the channel was 200 pS. The channel did not show any time-dependent inactivation and was observed to be constantly open. When Zn^2+^ was added to the pre-formed tolaasin channel, it completely inhibited channel activity, expectedly, because Zn^2+^ is a potent inhibitor of the tolaasin channel [5,6]. When TIF 16 was added to the cis chamber of the bilayer, channel activity was completely suppressed. Zn^2+^ and TIF 16 were used at concentrations of 1 mM and 30 µM, respectively. Although both Zn^2+^ and TIF 16 inhibited the tolaasin channel, the inhibitory effect of TIF 16 was shown to be nonreversible, as a washout with perfusing fresh buffer did not impede its effect.

### 2.5. Effect of TIF 16 on Oyster Mushroom Cultivation

**Figure 5:**
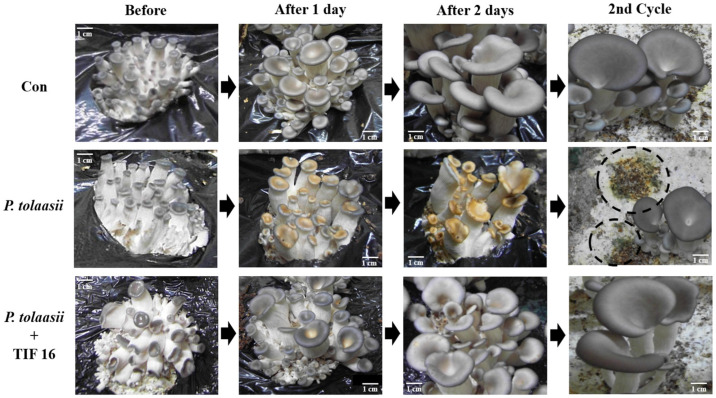
Suppression of brown blotch by TIF 16 during the shelf cultivation of oyster mushrooms. Disease was observed after 1 day of tolaasin treatment and became serious after 2 days. During the 2nd cycle of harvesting, fungi were spread on the surface of the cultivation shelf, as shown in the circles (*P. tolaasii*). Con, distilled water; *P. tolaasii*, *P. tolaasii* culture supernatant; *P. tolaasii* + TIF 16, *P. tolaasii* culture supernatant and TIF 16 at 10^−4^ M.

Oyster mushrooms were grown on cultivation shelves, and the effect of TIF 16 was measured at the early stage of fruiting body growth. Brown blotch disease was caused by treatment with the pathogen. The disease experiments were started and controlled when the size of the mushroom was less than 1 cm. After one day, the mushrooms treated with *P. tolaasii* pathogen showed inhibition of growth and ripening of fruiting bodies, resulting in dryness and shrinkage of the tissues (Figure 5, *P. tolaasii*). After two days, growth of the fruiting bodies of the treated mushrooms was abnormally stunted since the tolaasin molecules had caused disease. However, when the cultivated mushrooms were treated with TIF 16, the development of brown blotch was markedly reduced and normal growth of fruiting bodies was observed, compared with the mushrooms treated with only the pathogen (Figure 5, *P. tolaasii* + TIF 16).

To measure the persistence of the inhibitory effect of TIF 16, mushrooms grown in the first cycle were harvested from the experimental beds and the beds briefly washed with sterilized distilled water. To stimulate mushroom growth for a second cycle of cultivation, incubation was continued at 18 °C without any treatment. Only those mushrooms in the shelves originally treated with the *P. tolaasii* pathogens had their growth affected by persisting pathogens. Green molds were also observed growing on and contaminating the surface of the medium shelves. However, in the compartment containing mushrooms treated with both *P. tolaasii* and TIF 16, the cytotoxic effect of tolaasin was suppressed, and the growth of the mushrooms’ fruiting bodies remained healthy. Treatment with TIF 16 consistently protected the mushrooms from disease occurrence, even during the second cycle of growth.

## 3. Discussion

Although the cytotoxicity of tolaasin peptide has been investigated by various methods including hemolysis, pitting tests, and surface-tension studies [3,6,11], its working mechanisms have not been clearly verified. Pore-forming peptide toxins, such as tolaasin, melittin from bee venom, and mambalgins [13], reportedly are involved in several steps of cellular destruction processes of the cytoplasmic membrane. These peptide toxins bind to the host cell membrane, aggregate, and form membrane pores, followed by the collapse of transmembrane electrochemical gradients across the cytoplasmic membrane, affecting the flow of water, ions, and various metabolic molecules. Finally, the cells become swollen and lysed [6]. Brown blotch disease is also caused by the disturbance of intracellular osmotic pressure as excess cations move in and out through tolaasin channels.

In our previous study, various chemicals were selected from food additives as potential inhibitors of tolaasin toxicity. TIFs are able to interact with tolaasin molecules, probably through hydrophobic interactions directly or indirectly on the cytoplasmic membrane [11]. Among the various TIFs, TIF 16 was the most effective inhibitor that was selected from the food detergents. It is a polyglycerol ester of fatty acids that forms an ester with deca-glycerols and mono-myristate. The inhibitory effects of TIF 16 on tolaasin were observed in hemolysis, pitting tests, tolaasin channels formed in artificial lipid membranes, and shelf cultivation of oyster mushrooms. The effects of TIF 16 were measured under various temperature and pH conditions. Tolaasin cytotoxicity is affected by the structure and fluidity of the cytoplasmic membrane. Temperature is one of the major factors affecting cell membrane fluidity [14], and the cytotoxicity of tolaasin increases at higher temperatures. Inhibition of hemolytic activity by TIF 16 was observed across the temperature range of 17–47 °C (Figure 1). This indicates that the action of TIF 16 molecules is not affected by temperature. Tolaasin is an amphipathic peptide with both polar and non-polar regions [15], and the polarity of tolaasin molecules can be influenced by pH changes in the reaction solution. At pH 5, erythrocytes were very unstable, regardless of the presence of tolaasin, and rapid hemolysis was observed. Under neutral conditions (pH 6 and 7), the hemolytic activity of tolaasin was observed to be as usual, and 100% complete hemolysis was obtained within 30 min with 1 HU tolaasin. However, under alkaline conditions (pH 8 and 9), the hemolytic activity increased. These results are similar to those of a previous study showing that tolaasin binding to the cell membrane is promoted at alkaline pH values [11]. When the positive charges of tolaasin molecules decrease at alkaline pH values, tolaasin binding to the cytoplasmic membrane is increased, and, therefore, the hemolytic activity of tolaasin increases. Interestingly, TIF 16 effectively inhibited tolaasin-induced hemolytic activity under all pH conditions. The results showed that the inhibition of tolaasin cytotoxicity by TIF 16 was independent of temperature and pH, and thus TIF 16 could be suitable in various environmental conditions for practical and commercial applications.

The optimal concentration of TIFs to inhibit tolaasin toxicity is in the range of 10^−4^–10^−5^ M [11]. However, since TIFs from food detergents act as surfactants at high concentrations, the addition of TIFs promotes tolaasin-induced hemolysis at concentrations above 10^−3^ M [11]. Surfactants dissolve or disturb the lipid components of erythrocyte membranes; therefore, the addition of TIFs above a certain concentration to tolaasin-treated erythrocyte membranes can enhance the hemolytic activity. When tolaasin channels were formed and hemolysis of 10–20% completion had occurred on tolaasin-treated erythrocytes, the addition of 10^−5^ M TIF 16 immediately stopped further hemolysis (Figure 2). The inhibitory effects of TIF 16 were also measured after hemolysis was more than 40% complete, and the hemolytic curve showed a linear increase rather than a hyperbolic curve. These results indicate that TIF 16 can inhibit the preformed tolaasin channel. Although the working mechanism of TIF 16 is not known, the inhibitory effect of TIF 16 on the preformed tolaasin channel was confirmed (Figure 3). After tolaasin molecules were washed out from tolaasin-treated erythrocytes, a linear increase in hemolysis was observed before TIF 16 addition. After TIF 16 was added for 30 and 60 s and washed out from the reaction solution, inhibition of hemolysis was achieved, and furthermore, the inhibition was sustained. These results indicated that the inhibitory effect of TIF 16 was irreversible. Once TIF 16 binds to tolaasin-treated erythrocytes, it cannot be washed out. As TIF 16 inhibited tolaasin activity both before and after tolaasin channel formation, its effect on the pre-formed tolaasin channel was investigated. Possible ways to inhibit the virulent effects of pore-forming peptide toxins are by interfering with either their expression (inhibition of transcription regulators, quorum sensing) or interaction with a cognate receptor, structural modifications of membrane-bound precursors, or membrane insertion, with consequent transport of molecules or ions through formed pores [14,16,17]. Furthermore, some pore-forming toxins, such as melittin and pneumolysin, can be affected by the structure and dynamics of the membrane, such as its fluidity, rigidity, and thickness [18]. Tolaasin’s mode of action may be classified as of these mechanisms and, therefore, may influence the membrane structure or come to be affected by membrane fluidity. The effect of the erythrocyte concentration on hemolysis was evaluated and tolaasin-induced hemolysis was found to be mediated by a multi-hit process [19]. Furthermore, the membrane pore formation was explained by the aggregation of tolaasin molecules in the range of 2–6. This was investigated by gel permeation chromatography, HPLC, and SDS-PAGE analysis. The pore size of a tolaasin channel was determined to be 0.9 nm (9 Å) [19].

Tolaasin molecules formed two types of ion channels in the artificial lipid bilayer, and one of them had two sub-conductance states. Membrane currents were greatly increased and the lipid bilayer became unstable (leaky membrane) with the addition of tolaasin at a higher concentration than 0.8 HU. The optimum concentration of tolaasin for monitoring the single channel current was 0.6 HU [5]. The number of tolaasin molecules required to form a tolaasin channel was calculated as ten. Since one tolaasin molecule is able to form three turns of the α-helix structure in the membrane, there are two pentamers, one in the outside- and the other in the inside-leaflet of the membrane bilayer, meaning a six-turn α-helix transmembrane segment can be formed. When two pentamers present inside and outside the membrane leaflet are overlap matched, it is expected that a complete channel is formed and the square-shaped currents of a single channel are shown; however, when these pentamers are misaligned, the tolaasin channel is closed [19]. In the artificial lipid bilayer, tolaasin channels were formed, and the inhibitory effect of TIF 16 on the tolaasin channels was confirmed (Figure 4). The tolaasin channels exhibited a unitary conductance of 220 pS with long-lasting openings. TIF 16 rapidly and irreversibly inhibited the tolaasin channels, similar to Zn^2+^. This result confirmed that TIF 16 inhibited the formation of tolaasin channels. Tolaasin channels were inhibited by Zn^2+^ and TIF 16 at concentrations of 1 mM and 30 µM, respectively. TIF 16 showed an inhibitory effect at a concentration about 1/30 times lower than that of Zn^2+^. This indicated that tolaasin channels were more sensitive to TIF 16 than to Zn^2+^; therefore, TIF 16 was shown to be a potent inhibitor of tolaasin channels. Zn^2+^ blocks the channels through mouse plugging of tolaasin pores in the cytoplasmic membrane [5], while TIF 16 seems to suppress the tolaasin channels via a different mechanism. Two working models are possible: blocking channels by direct binding to tolaasin channels, or inhibiting tolaasin channel formation by disturbing the lipid bilayer.

The ability of TIF 16 to control brown blotch disease was evaluated in on-farm oyster mushroom cultivation. Based on our results, TIF 16 was highly effective in preventing the disease and delaying its progression. While tolaasin molecules collapsed mushroom tissue and formed blotches on the surface of mushrooms, TIF 16 at 10^−4^ M completely suppressed the cytotoxicity of tolaasin; no symptoms of disease were observed, and on-farm cultivation was successful (Figure 5). On treatment with TIF 16, tolaasin toxicity was effectively inhibited in a dose-dependent manner [11]. At even slightly higher concentrations of TIF 16, no damage to the mushrooms was observed, and normal growth of mushrooms was measured during shelf cultivation. Since a long-term feeding study in rats showed no adverse effects at 5% polyglycerol ester of fatty acid (PEFA) (corresponding to 2500 mg/kg bw per day), it is expected that the concentration of TIF 16 (10^−4^ M) may not cause problems to other organisms and workers in the cultivation house and environments [20].

In a similar case to TIF 16, melittin, the main toxic component of honeybee venom, was found to be sensitive to solution conditions (ionic strength, pH, solution concentration) and it had an α-helical structure in methanol solution [21,22]. These toxic peptide monomers can self-assemble to form a tetramer, which becomes a transmembrane pore on cell membranes. Previously, there were limited studies on melittin inhibitors, such as peptide- and polymer-based inhibitors [23]. Kanemitsu et al. [24] found that 5-chlorotryptamine (5-CT) acted as an inhibitor of melittin-induced hemolysis. Fluorescence quenching, circular dichroism measurements, and size-exclusion chromatography revealed that 5-CT interacted with Trp19 in melittin and affected the formation of the melittin tetramer involved in hemolysis.

In conclusion, various methods have been reported for the prevention or control of brown blotch disease during the cultivation of mushrooms; however, none have enabled complete control of the disease. Tolaasin-inhibitory factors (TIFs) have been found effective, and in our study, the most effective compound was TIF 16. It could completely suppress tolaasin-induced hemolysis and tolaasin channels in artificial lipid bilayers and showed an excellent inhibitory effect on tolaasin cytotoxicity. TIF 16 is a polyglycerol fatty acid ester molecule obtained from food additives; therefore, it is considered a good candidate for the prevention of brown blotch disease during the cultivation of mushrooms.

## 4. Materials and Methods

### 4.1. Purification of Tolaasin and Its Hemolytic Activity

Tolaasin was purified using the method described by Cho et al. [5] with minor modifications. Tolaasin molecules in the culture supernatant of *P. tolaasii* 6264 (GenBank Accession Number: JN187439) were collected by ammonium sulfate precipitation and ultracentrifugation (XL-70; Beckman Instruments, Inc., Palo Alto, CA, USA). After the precipitated crude tolaasin was suspended in 10 mM sodium phosphate buffer (pH 7.0), it was dialyzed in the same buffer for 4 h, homogenized, and stored in a deep freezer (−70 °C).

The hemolytic activity of tolaasin was measured in rat erythrocytes (DBL, Eumseong, Republic of Korea). Defibrinated rat erythrocytes were washed with HEPES-buffered saline (HBS; 5 m M HEPES (N-(2-hydroxyethyl)piperazine-N’-(2-ethanesulfonic acid), 150 mM NaCl, 5 mM KCl, and 1 mM MgSO_4_, pH 7.4), and thereafter diluted with the same buffer to obtain a 10% solution. Tolaasin diluted with HBS was added to the erythrocyte reaction solution and incubated at 37 °C. Hemolysis was monitored by measuring absorbance changes at 600 nm using a UV/Vis spectrophotometer (U-2000, Hitachi Ltd., Tokyo, Japan). One hemolytic unit (HU) of tolaasin was defined as the amount of tolaasin able to induce complete hemolysis of 1% erythrocytes within 30 min. The concentration of TIF 16 (SY-GLYSTER MM-750, Sakamoto Yakuhin Kogyo, Osaka, Japan) was 10^−5^ M for the hemolysis experiments. Buffer solutions with pH values ranging from 4 to 9 were prepared as follows: solution of pH 5 with 30 mM acetic acid buffer and saline solution; solutions of pH 6 and 7 with 30 mM HBS; and solutions of pH 8 and 9 with 30 mM Tris buffer.

### 4.2. Effect of TIF 16 on Tolaasin Channel

Ion channel formation by tolaasin was measured using the method described by Zakharian [25]. Briefly, a planar lipid bilayer, composed of phosphatidyl ethanolamine and phosphatidyl serine dissolved in *n*-decane (20 mg/mL) at a 1:1 ratio, was formed on a hole of 0.25 mm diameter made in a Delrin cup. Tolaasin was added to the *cis* chamber containing 100 mM KCl and 10 mM HEPES (pH 7.4). Channel recordings were measured under symmetric ionic conditions using the same buffer as the solution in the *trans* chamber. The solution in the *cis* chamber was connected to the bilayer via an Ag/AgCl electrode and an agar/KCl bridge and by recording the head-stage input of an Axopatch 1D amplifier (Axon Instruments, Foster City, CA, USA). The solution in the *trans* chamber was held at ground potential using the same electrode arrangement. Membrane currents were amplified with an Axopatch 1D amplifier and recorded on a VCR tape using a video tape recorder (SV-8200D, Samsung Electronics Co., Ltd., Suwon, Republic of Korea). The recorded current signals were filtered through a low-pass Bessel filter at 1 kHz (Dagan Corporation, Minneapolis, MN, USA). The filtered signals were digitized at 10 kHz by an Axolab 1100 interface (Axon Instruments, Foster City, CA, USA). Single-channel currents were analyzed using pClamp software (version 8.01, Axon Instruments). When the tolaasin channel was formed, the inhibitory effects of Zn^2+^ and TIF 16 were recorded and analyzed by adding 1 mM ZnCl_2_ and 30 mM TIF 16, respectively.

### 4.3. Effects of TIF 16 on Oyster Mushroom Cultivation

Seedlings of oyster mushrooms were cultivated in 1 m wide and 2 m long beds filled with sterilized sawdust medium. After three weeks, once the beds were fully covered with mycelia, the temperature in the cultivation house was lowered to below 18 °C to induce development of the fruiting body. The cultivation bed was divided into three sections: control, *P. tolaasii* (culture supernatant), and *P. tolaasii*+TIF 16. Each treatment was sprayed 2–3 times on the fruiting bodies. TIF 16 of 10^−4^ M was used, as this was the concentration found not to affect the growth of the fruiting bodies. Acrylic plate walls with a 20 cm height were installed to minimize disturbance between the three sections.

## Data Availability

The data and analyses from the current study are available from the corresponding author on reasonable request.

## References

[1] Tolaas A.G. (1915). A bacterial disease of cultivated mushrooms. Phytopathology.

[2] Peng J.T. (1986). Resistance to Disease in *Agaricus bisporus* (lange) Imbach. Ph.D. Thesis.

[3] Hutchison M.I., Johnstone K. (1993). Evidence for the involvement of the surface active properties of the extracellular toxin tolaasin in the manifestation of brown blotch disease symptoms by *Pseudomonas tolaasii* on *Agaricus bisporus*. Physiol. Mol. Plant. Pathol..

[4] Otsuka Y., Ishikawa T., Takahashi C., Masuda M. (2019). A short peptide derived from the ZorO toxin functions as an effective antimicrobial. Toxins.

[5] Cho K.H., Kim Y.K. (2003). Two types of ion channel formation of tolaasin, a *Pseudomonas* peptide toxin. FEMS Microbiol. Lett..

[6] Rainey P.B., Brodey C.L., Johnstone K. (1991). Biological properties and spectrum of activity of tolaasin, a lipodepsipeptide toxin produced by the mushroom pathogen *Pseudomonas tolaasii*. Physiol. Mol. Plant. Pathol..

[7] Wong W.C., Preece T.F. (1985). *Pseudomonas tolaasii* in cultivated mushroom (*Agaricus bisporus*) crops: Effects of sodium hypochlorite on the bacterium and on blotch disease severity. J. Appl. Microbiol..

[8] Geels F.P., van Griensven L.D., Rutjens A.J., Maher M.J. (1991). Science and Cultivation of Edible Mushrooms. Mushroom Science XIII.

[9] Tajalipour S., Hassanzadeh N., Jolfaee H.K., Heydari A., Hasemi A. (2014). Biological control of mushroom brown blotch disease using antagonistic bacteria. Biocontrol. Sci. Technol..

[10] Geels F.P. (1995). *Pseudomonas tolaasii* control by kasugamycin in cultivated mushroom (*Agaricus bisporus*). J. Appl. Microbiol..

[11] Yun Y.B., Kim M.H., Han J.H., Kim Y.K. (2017). Suppression of brown blotch disease by tolaasin inhibitory factors. J. Appl. Biol. Chem..

[12] Kim S.T., Choi T.K., Kim Y.K. (2007). pH-dependent cytotoxicity of a peptide toxin, tolaasin. J. Korean Soc. Appl. Biol. Chem..

[13] Sun D., Yu Y., Xue X., Pan M., Wen M., Li S., Qu Q., Li X., Zhang L., Li X. (2018). Cryo-EM structure of the ASIC1a-mambalgin-1 complex reveals that the peptide toxin mambalgin-1 inhibits acid-sensing ion channels through an unusual allosteric effect. Cell Discov..

[14] Shewell L.K., Harvey R.M., Higgins M.A., Day C.J., Hartley-Tassell L.E., Chen A.Y., Gillen C.M., James D.N.A., Alonzo F., Torres V.J. (2014). The cholesterol-dependent cytolysins pneumolysin and streptolysin O require binding to red blood cell glycans for hemolytic activity. Proc. Natl. Acad. Sci. USA.

[15] Soler-Rivas C., Moller A.C., Arpin N., Olivier J.M., Wichers H.J. (2001). Induction of a tyrosinase mRNA in *Agaricus bisporus* upon treatment with a tolaasin preparation from *Pseudomonas tolaasii*. Physico. Mol. Plant Pathol..

[16] Statt S., Ruan J.W., Hung L.Y., Chang C.Y., Huang C.T., Lim J.H., Li J.D., Wu R., Kao C.Y. (2015). Statin-conferred enhanced cellular resistance against bacterial pore-forming toxins in airway epithelial cells. Am. J. Respir. Cell Mol. Biol..

[17] Omersa N., Podobnik M., Anderluh G. (2019). Inhibition of pore-forming proteins. Toxins.

[18] Ganpule S., Vijaya A.K., Sukova A., Preta G. (2022). Membrane cholesterol content and lipid organization influence melittin and pneumolysin pore-forming activity. Toxins.

[19] Cho K.H., Wang H.S., Kim Y.K. (2010). Temperature-dependent hemolytic activity of membrane pore-forming peptide toxin, tolaasin. J. Pept. Sci..

[20] Younes M., Aggett P., Aguilar F., Crebelli R., Dusemund B., Filipic M., Frutos M.J., Galtier P., Gott D., EFSA Panel on Food Additives and Nutrient Sources added to Food (EFSA ANS Panel) (2017). Scientific Opinion on the re-evaluation of polyglycerol esters of fatty acids (E 475) as a food additive. EFSA J..

[21] Raghuraman H., Chattopadhyay A. (2007). Melittin: A membrane-active peptide with diverse functions. Biosci. Rep..

[22] Klocek G., Schulthess T., Shai Y., Seelig J. (2009). Thermodynamics of melittin binding to lipid bilayers, aggregation and pore formation. Biochemistry.

[23] Mihajlovic M., Lazaridis T. (2010). Antimicrobial peptides in toroidal and cylindrical pores. Biochim. Biophys. Acta Biomembr..

[24] Kanemitsu S., Morita K., Tominaga Y., Nishimura K., Yashiro T., Sakurai H., Yamamoto Y., Kurisaki I., Tanaka S., Matsui M. (2022). Inhibition of melittin activity using a small molecule with an indole ring. J. Phys. Chem. B.

[25] Zakharian E. (2013). Recording of ion channel activity in planar lipid bilayer experiments. Methods Mol. Biol..

